# Effects of Lifestyle Changes on the Mental Health of Healthcare Workers with Different Sense of Coherence Levels in the Era of COVID-19 Pandemic

**DOI:** 10.3390/ijerph18062801

**Published:** 2021-03-10

**Authors:** Kento Tanaka, Masatoshi Tahara, Yuki Mashizume, Kayoko Takahashi

**Affiliations:** 1 Graduate School of Medical Sciences, Kitasato University, Kanagawa 252-0373, Japan; dm19017@st.kitasato-u.ac.jp (M.T.); dm18026@st.kitasato-u.ac.jp (Y.M.); 2 Department of Rehabilitation Therapist, Saiseikai Higashikanagawa Rehabilitation Hospital, Kanagawa 221-0822, Japan; 3 Department of Occupational Therapy, School of Allied Health Sciences, Kitasato University, Kanagawa 252-0373, Japan

**Keywords:** COVID-19, mental health, sense of coherence, lifestyle, healthcare worker

## Abstract

Sense of coherence (SOC) is a psychological factor that contributes to mental health maintenance under stressful environment. Likewise, level of SOC might affect mental health among healthcare workers during the COVID-19 pandemic differently. In this study, we investigated the relationships between lifestyle changes and mental health (General Health Questionnaire-12: GHQ-12) among different level of SOC (weak, moderate, or strong by SOC-13). The data of 898 healthcare workers from cross-sectional survey dataset were extracted and analyzed. As results, based on GHQ-12 score, 86.1% of 244 participants with weak SOC, 60.1% of 606 participants with moderate SOC, and 31.3% of 48 participants with strong SOC had poor mental health. Both SOC levels and lifestyle changes (except alcohol consumption) had significant main effects on the GHQ-12 score. Analysis on the association between lifestyle changes and mental health status stratified by SOC level reveled that among participants with weak SOC, those who increased their leisure and activity time had reduced odds of poor mental health than those who made no changes (OR: 0.08, CI: 0.01 to 0.64). Healthcare workers with weak SOC were at risk of poor mental health during the COVID-19 pandemic, and lifestyle changes may improve their mental health.

## 1. Introduction

The coronavirus disease 2019 (COVID-19) pandemic has resulted in significant impacts on daily living. In addition to a fear of infection, mental health issues have arisen. Studies have shown that healthcare workers on the front line had increased mental health issues [1]. Moreover, Tahara et al., [2] reported that healthcare workers who were not on the front line had higher rate of severe mental health compared to general population even though their work have not changed. Therefore, concerns regarding mental health problems during COVID-19 apply to all healthcare workers. The COVID-19 pandemic has also resulted in numerous lifestyle changes. The governments of many countries declared nationwide quarantines or implemented emergency measures, such as lockdowns, to slow the spread of COVID-19. In Japan, the Ministry of Health, Labor, and Welfare announced a “new lifestyle” for people to practice in their daily lives to prevent infections [3]. Recent surveys have indicated changes in physical activity levels, sleep quality, and exercise frequency compared with pre-COVID-19 levels [4,5]. Lifestyle changes caused by environmental factors can negatively affect mental health [6,7].

Although many stressful and drastic changes occur, an individual’s sense of coherence (SOC) can be a critical factor for maintaining mental and physical health. Antonovsky [8] developed the concept of SOC in his Salutogenesis theory, which focuses on factors that contribute to health rather than focusing on the stressor. In this theory, SOC describes how well people cope with stressors. SOC is composed of three dimensions: manageability, comprehensibility, and meaningfulness. Manageability reflects an individual’s perception that they have access to the necessary resources to meet the various demands imposed by stressful situations and events. Comprehensibility represents an individual’s perception that situations and events are structured and clear. Meaningfulness describes an individual’s perception of the demands and challenges in life that are worth investment and engagement.

From a mental health perspective, previous studies have shown that level of SOC explains the level of psychological distress [9], and individuals with weak SOC had a higher risk of experiencing mental health problems than those with moderate or strong SOC [10]. Therefore, improving SOC could help individuals maintain their mental health during the pandemic. However, studies that have shown SOC improvements used long-term interventions (four months or longer), whereas short-term improvements in SOC have been minimal [11,12]. Furthermore, the current dogma regarding SOC development is that “SOC is a stable disposition after 30 years of age” [8].

Many studies investigating the effects of SOC have emphasized the importance of having strong SOC. However, no study has recommended lifestyle changes for individuals with different SOC strengths to improve their abilities to cope with stress and maintain good mental health, despite the widespread acknowledgment that SOC strength varies among individuals. Our hypotheses are that when healthcare works are trichotomized into different levels of SOC, people in weak SOC level would likely to have severe mental health issue compared to people with modern or strong SOC levels. We also hypothesized people in weak SOC level uses different strategies such as a lifestyle change to maintain mental health in this stressful environment.

Therefore, this study’s objectives were to: (1) investigate the mental health status associated with varying SOC levels and (2) explore the associations between lifestyle changes and mental health during the COVID-19 pandemic among healthcare workers stratified by SOC level.

## 2. Materials and Methods

### 2.1. Design, Sample, and Setting

This study was a secondary analysis using the database generated by Tahara et al. [2]. The database was generated following a cross-sectional survey that was performed using a web-based questionnaire (Google Form^®^) from 30 April 2020, to 6 May 2020. The survey used a snowball recruitment method, starting with alumni of the occupational therapy program at Kitasato University, and 929 people were registered. In this study, the database included responses from healthcare workers: occupational therapists, physical therapists, physicians, nurses, and other healthcare workers (occupational therapy assistants, speech and language pathologists, radiological technologists, nutritionists, caretakers, pharmacists, occupational health nurses, psychologists, medical technologists, dentists, and social workers). Other occupations were excluded. The original study was approved by the Ethical Review Board of the School of Allied Health Sciences, Kitasato University (#2020-013D). And all participants were informed and consented for the data to be used in the secondary analysis.

### 2.2. Measurement

#### 2.2.1. Sociodemographic Variables

The following variables, which are thought to be associated with mental health, were collected: age, gender, occupation, economic status, and the number of people in the household. The number of people in the household was divided into two categories, 1: alone and 2: with others, and the variable was defined as living arrangement. Economic status was also divided into two categories, such that the original categories, 1: not affordable; 2: not much affordable; 3: affordable; and 4: very affordable, became 1: affordable and 2: not affordable.

#### 2.2.2. Sense of Coherence (SOC)

The 13-item Sense of Coherence scale (SOC-13), which was initially developed by Antonovsky [13] and translated into Japanese by Yamazaki [14] was used to measure SOC. This measure consists of 13 items rated using a 7-point Likert scale (1: very often to 7: rarely or never), with total scores ranging from 13 to 91, Cronbach’s alpha coefficient for the scale was 0.77 for this study. In this study, SOC was used as a trichotomized variable, divided into weak, moderate, and strong SOC. Trichotomization was performed to facilitate the comparison of groups who are regarded to have weak or strong SOC in the general population. Trichotomization was conceptually introduced by Antonovsky and has been applied in other research [15,16,17]. We categorized the SOC levels using the national average (NA: 58.99) and standard deviation (SD: 12.18) for SOC-13 scores that were previously reported for a nationwide study performed in Japan [18]. To improve the generalizability of the study results, participants with SOC scores lower than or equal to one standard deviation below the NA (SOC ≤ 46.81) were categorized as having weak. Participants with SOC scores higher than or equal to one standard deviation above the NA (SOC ≥ 71.16) were categorized as having strong SOC. All others were categorized as having moderate SOC.

#### 2.2.3. Mental Health Status

Mental health was measured using the Japanese version of the General Health Questionnaire-12 (GHQ-12) [19,20], which consists of 12 questions regarding their feelings over the past few weeks. The GHQ-12 is used as a screening tool to detect nonpsychotic psychiatric diseases and to evaluate general mental health. The GHQ method (0-0-1-1 point allocation) was used to calculate the GHQ-12 scores [21] GHQ-12 scores can range from 0 to 12, with higher scores indicating increased psychological distress. We defined a GHQ-12 score ≥ 4 as poor mental health and a score < 4 as healthy mental health [21]. Cronbach’s alpha coefficient of this measurement was 0.79.

#### 2.2.4. Lifestyle Changes during the COVID-19 Pandemic

Lifestyle change categories included the following: communication with friends, communication with family, leisure and activity, exercise, food intake, sleep duration, and alcohol consumption. Each individual was asked whether their lifestyle for each category was “decreased from usual”, “same as usual”, or “increased from usual”. For alcohol consumption, we added the alternative answer, “non-drinker”.

### 2.3. Data Analysis

#### 2.3.1. Sample Characteristics

Differences associated with gender, living arrangement, economic status, and employment status were analyzed by Fisher’s exact test, and percentages were calculated. Because occupation had many categories, the degrees of freedom were too large for Fisher’s exact test to be performed; instead, the Chi-square test was used. For differences associated with SOC-13 scores and age, analysis of variance (ANOVA) was conducted.

#### 2.3.2. Mental Health among Healthcare Workers with Different SOC Levels during the Pandemic

Descriptive statistics were performed for the GHQ-12 scores, SOC-13 scores, and mental health status (GHQ-12 score ≥ 4: poor mental health, GHQ-12 score < 4: good mental health) to calculate the mean and standard deviation or number and frequency (%). Differences in GHQ-12 scores were analyzed by ANOVA, followed by Tukey’s honestly significant difference (HSD) test for multiple comparisons. Mental health status was analyzed using Chi-square tests with Bonferroni correction for multiple comparisons.

#### 2.3.3. Associations between Lifestyle Changes and Mental Health during the COVID-19 Pandemic among Healthcare Workers according to SOC

A two-way ANOVA was used to investigate the main effects and the interaction effect of SOC level and each lifestyle change on mental health. Eta squared (η^2^) was used to investigate the effect size.

A logistic regression model was used to calculate the odds ratios (ORs) and 95% confidence intervals (95% CIs) for the risk of poor mental health (GHQ-12 score ≥ 4) associated with each lifestyle change in each SOC category. The dependent variable was the dichotomized value of the GHQ-12, and the independent variables included the seven lifestyle changes, and other variables were used to adjust for confounding effects, including age, gender, living arrangement, and economic status. For each lifestyle change, multiple categorical variables were created, with the “same as usual” response set as the reference category.

*p*-values of 0.05 or less were considered significant. Analyses were performed using JMP PRO^®^ software, version 14 (SAS Institute, Cary, NC, USA).

## 3. Results

### 3.1. Sample Characteristics

The data for 898 healthcare workers were collected from the database. 31 people were excluded from the study since their occupation (i.e., university faculty and engineers) could not be defined to be related to healthcare field. Of these, 244 (27.2%) individuals were categorized as weak SOC, 606 (67.5%) were classified as moderate SOC, and 48 (5.3%) were categorized as strong SOC. The mean SOC-13 scores were significantly different between each SOC category. Table 1 presents the differences in age, gender, living arrangement, economic status, and occupation according to the SOC level. Age, occupation, and employment status did not differ significantly across SOC levels. However, other characteristics were significantly different between SOC levels (gender: *p* = 0.002; living arrangement: *p* < 0.001; economic status: *p* < 0.001; GHQ-12 cutoff: *p* < 0.001; GHQ-12 score: *p* < 0.001; SOC-13 score: *p* < 0.001). Those with weak SOC were more likely to be male, living alone, and not in an acceptable economic situation.

### 3.2. Mental Health Status among Healthcare Workers according to SOC Levels

Table 2 differences in GHQ-12 scores and the percentages of individuals with poor mental health status according to each SOC level during the COVID-19 pandemic. Among individuals with weak SOC, 86.1% had poor mental health, 60.1% of individuals with moderate SOC had poor mental health, and 31.3% of individuals with strong SOC had poor mental health. Both GHQ-12 scores and dichotomized mental health status were significantly different across SOC levels (*p* < 0.001 and *p* < 0.001, respectively). Multiple comparisons revealed that lower SOC levels were associated with significantly lower GHQ-12 scores and a higher rate of poor mental health.

### 3.3. Associations between Lifestyle Changes and Mental Health during the COVID-19 Pandemic among Healthcare Workers with Varying SOC Levels

Table 3 shows the mean and standard deviation of the GHQ-12 score, according to lifestyle changes and SOC levels. The two-way ANOVA showed a significant main effect of SOC level on the GHQ-12 score (*p* < 0.01). Lifestyle changes, including communication with friends, communication with family, leisure and activity, exercise, food intake, and sleep duration, had significant main effects on the GHQ-12 score (*p* = 0.006, 0.021, 0.011, 0.011, 0.020, 0.002, respectively). However, no main effect of alcohol consumption was observed on the GHQ-12 score. No significant interactions were observed between the SOC level and any lifestyle change.

Table 4 presents the association between poor mental health (GHQ-12 score ≥ 4), including ORs and 95% CI values, and each SOC level. Among people with weak SOC, increased leisure and activity was related to a decreased risk of poor mental health (OR: 0.08; 95% CI: 0.01 to 0.64; *p* = 0.015). Decreased sleep duration was associated with a increased risk of poor mental health (*p* = 0.015); however, because 34 people who reported decreased sleep duration were all defined as having poor mental health, the odds ratio was 2.46 × 10^8^, and the CI could not be calculated. Among individuals with moderate SOC, the following lifestyle changes were associated with increased odds of poor mental health status compared with those who did not make lifestyle changes: decreased communication with friends (OR: 2.41; 95% CI: 1.59 to 3.65; *p* < 0.001); and decreased leisure and activity (OR: 1.63; 95% CI: 1.05 to 2.53; *p* = 0.031); decreased sleep duration (OR: 3.39; 95% CI: 1.45 to 7.93; *p* = 0.002). Individuals who did not change their alcohol consumption amounts had a lower risk of poor mental health than individuals who did not consume alcohol (OR: 0.64; 95% CI: 0.40 to 0.96; *p* = 0.030). Among those individuals with strong SOC, those who increased their sleep consumption were more likely to have poor mental health (OR: 404.15; 95% CI: 2.67 to 61,182; *p* = 0.030). Other lifestyle changes among the various SOC categories were not significantly associated with mental health status.

## 4. Discussion

### 4.1. Sample Characteristics

The most common healthcare workers in this study were occupational therapists. In Japan, the average age of an occupational therapist is 34.6 years, and 68% are women [22], which accounts for the high rate of women in our study and the young age of study participants relative to that of the general population. In this study, healthcare workers had a lower mean SOC-13 score than that reported for a national study in Japan (Our study, mean: 52.9; National study, mean: 58.99) [18]. Therefore, the number of people classified as having strong SOC was smaller than expected compared with the number classified as having weak SOC. This result may indicate that healthcare workers tend to have weaker SOC than the general population (Nurses, median: 50, IQR: 45 to 55; occupational therapists, median: 53, IQR: 47.0 to 60.8) [23,24] or may suggest that the SOC of this population weakened during this pandemic [25].

Differences were observed in gender, economic status, and living arrangements among the three SOC groups. The relationship between SOC and gender remains under debate, as some studies have reported significant differences between men and women, while others have stated that the level does not depend on gender [26,27]. The observed differences in economic status and living arrangements between SOC levels in this study were consistent with those reported by a previous study [28].

### 4.2. Mental Health Status for Each SOC Level during the COVID-19 Pandemic

A previous study reported that 40.4% of the general population had poor mental health during the pandemic, as assessed using the GHQ-12, which was also used in the present study [29]. Before the COVID-19 pandemic, 29.0% of Japan’s general population was reported to have poor mental health [30]. Our results showed that 65.6% of healthcare workers had poor mental health. Moreover, 86.1% of individuals classified as having weak SOC had poor mental health, which was a larger proportion than those with moderate (60.1%) and strong (31.3%) SOC. Therefore, healthcare workers, especially those with weak SOC, are thought to be at higher risk of poor mental health than the general population.

These findings match those of another study that reported that people with weak SOC had a higher risk of mental health issues, both during and before the pandemic [9,10]. Antonovsky stated that health status could influence SOC. However, recent papers have reported that SOC is a causal variable, moderator, or mediator of health status [31,32], and could not be predicted by other psychological factors [33]. The effects of SOC on mental health are thought to act through two mechanisms. One mechanism is that individuals with stronger SOC perceived less stress associated with daily living [34,35]. The second mechanism is that SOC may moderate stressors [31,32]. During the COVID-19 pandemic, which introduced strong stressors for both daily living and in healthcare workplaces, healthcare workers with weak SOC should pay particular attention to their own mental health status.

### 4.3. Associations between Lifestyle Changes and Mental Health during the COVID-19 Pandemic among Healthcare Workers with Varying SOC Levels

The SOC level and all lifestyle changes, except for alcohol consumption, had main effects on mental health. Although a large effect size was observed for the association between mental health and SOC, lifestyle changes were also associated with changes in mental health. The analysis according to SOC level showed different patterns for the associations between lifestyle changes and mental health. Changing one’s SOC may be difficult for those older than 30 years or when in a chaotic environment, such as during a pandemic. Therefore, lifestyle changes might help individuals with weak SOC maintain their mental health during pandemic conditions.

The results observed for healthcare workers with a moderate SOC might be applicable to the general population. Among healthcare workers with moderate SOC, an association was observed between decreased communication with friends and poor mental health, which may be associated with the feeling of loneliness reported by another study during the pandemic [36]. The association between decreased sleep duration and worsen mental health was expected. Healthcare workers who did not change their alcohol consumption pattern were less likely to be classified as having poor mental health compared with those who did not consume alcohol, which is similar to the results reported by other studies [37,38].

Healthcare workers with weak SOC should be aware of the effects of changing lifestyles compared with healthcare workers with moderate or strong SOC because those with weak SOC are at higher risk of poor mental health. Among healthcare workers with weak SOC, increasing their leisure and activity time (such as shopping, yoga, muscle training, et at.), and avoiding a decrease in sleep time could help maintain their mental health status. Because our results did not show any significant interaction effects between the SOC level and any lifestyle changes on GHQ-12 scores, we cannot emphasize that an increase in leisure and activity time among individuals with weak SOC may be associated with mental health outcomes. However, several studies have found that leisure and activity are associated with psychological health [39,40]. Combined with the results for the moderate SOC group, which suggested that decreased leisure and activity was associated with poor mental health, we suggest that people find a way to ensure adequate leisure and activity time during stressful periods, such as the pandemic.

### 4.4. Limitations

This study had several limitations that should be considered when interpreting the results. First, this study was a secondary data analysis of data from a cross-sectional survey. Thus, these conclusions are limited to associations and causation could not be determined, also the results could not be referred to the impact of COVID-19 alone. Second, because this was a secondary analysis of existing data, some factors that are known to be related to mental health and SOC could not be included in the analysis. For example, educational level, job-related stress, and the date when the survey was conducted could be confounding factors that affect the association between lifestyle changes and mental health. Third, due to the imbalances in the sizes of the SOC groups, the weak and strong SOC groups had small sample sizes, resulting in wide confidence intervals, which made determining associations between lifestyle changes and mental health in these two groups difficult.

### 4.5. Strengths of Our Study

To our knowledge, this is the first study that investigates the mental health status associated with varying SOC levels during the COVID-19 pandemic. Because little is known about the associations between lifestyle and mental health according to the SOC level, our study adds a new perspective that can support the mental health of healthcare workers.

## 5. Conclusions

In conclusion, we found that mental health status differed across SOC levels during the COVID-19 pandemic and revealed that the majority of individuals with weak SOC had poor mental health. Lifestyle changes were associated with changes in mental health across all SOC levels. Among healthcare workers with weak SOC, increased leisure and activity and decreased sleep duration were the lifestyle changes most strongly associated with changes in mental health. Future studies should focus on people with weak SOC and investigate factors that could help them maintain their mental health.

## Figures and Tables

**Table 1:** Demographic characteristics of study participants (*n* = 898).

SOC Level	Overall	Weak SOC		Moderate SOC		Strong SOC			*p-*Value
Variables	*n* = 898	*n* =244		*n* = 606		*n* = 48			
SOC-13 (mean ± SD)	52.9	±11.1	39.6	±5.5	56.4	±6.4	76.5	±3.8	0.001 ^(1)^
Age (mean ± SD)	32.1	±8.1	31.3	±7.9	32.3	±8.0	33.3	±9.0	0.133 ^(1)^
Gender *n* (%)									
Male	385	(42.9%)	88	(36.1%)	267	(44.1%)	30	(62.5%)	0.002 ^(2)^
Female	513	(57.1%)	156	(63.9%)	339	(55.9%)	18	(37.5%)	
Living arrangement *n* (%)									
With others	663	(73.8%)	159	(65.2%)	464	(76.6%)	40	(83.3%)	0.001 ^(2)^
Alone	235	(26.2%)	85	(34.8%)	142	(23.4%)	8	(16.7%)	
Economic status *n* (%)									
Acceptable	583	(64.9%)	135	(55.3%)	415	(68.5%)	33	(68.8%)	0.001 ^(2)^
Not acceptable	315	(35.1%)	109	(44.7%)	191	(31.5%)	15	(31.3%)	
Jobs *n* (%)									
Occupational therapist	650	(72.4%)	184	(81.8%)	435	(81.6%)	31	(64.6%)	0.122 ^(3)^
Physical therapist	181	(20.2%)	42	(18.7%)	125	(23.5%)	14	(29.2%)	
Speech therapist	24	(2.7%)	6	(2.7%)	18	(3.4%)	0	(0%)	
Nurse	13	(1.8%)	4	(1.8%)	9	(1.7%)	0	(0%)	
Medical doctor	12	(1.3%)	2	(0.9%)	7	(1.3%)	3	(5.2%)	
Other	18	(2.0%)	6	(2.5%)	12	(2.0%)	0	(0%)	

SOC level: Weak SOC (SOC-13 ≤ 46), Moderate SOC (46 < SOC-13 < 72), Strong SOC (SOC-13 ≥ 72). GHQ: General Health Questionnaire, SOC: Sense of coherence, SD: standard deviation. ^(1)^ *p* value from analysis of variance, ^(2)^ *p* value from Fisher’s exact test, ^(3)^ *p* value from Chi-square test.

**Table 2:** Mental health measurements according to sex, clinical workplace phase, and place of residence (*n* = 898).

SOC Level	Overall		Weak SOC		Moderate SOC		Strong SOC		*p* Value
Variables	*n* = 898		*n* = 244		*n* = 606		*n* = 48		
Total GHQ-12 (mean ± SD)	5.07 ± 3.1		6.97 ± 2.9 *^,†^		4.50 ± 2.95 ^†^		2.58 ± 2.4		<0.001 ^(1)^
Mental health status *n* (%)									
Poor (GHQ-12 ≥ 4)	589	(65.6%)	210	(86.1%) 5 *^,†^	364	(60.1%) ^†^	15	(31.3%)	<0.001 ^(2)^
Healthy (GHQ-12 < 4)	309	(34.4%)	34	(13.9%)	242	(39.9%)	33	(68.8%)	

SOC level: Weak SOC (SOC-13 ≤ 46); Moderate SOC (46 < SOC-13 < 72); Strong SOC (SOC-13 ≥ 72). GHQ: General Health Questionnaire, SOC: Sense of coherence; SD: standard deviation. * Significant compared with the moderate SOC, ^†^ Significant compared with the strong SOC. ^(1)^ *p*-value from analysis of variance, ^(2)^ *p*-value from Chi-square test.

**Table 3:** Mean GQH-12 scores according to SOC level and *p*-values of main and interaction effects.

SOC Level		Weak SOC		Moderate SOC		Strong SOC		*p*, η^2^		
Variables		*n*	Mean ± SD	*n*	Mean ± SD	*n*	Mean ± SD	SOC Level	Lifestyle	Interaction
Communication with friends	decreased	153	7.25 ± 0.23	379	4.98 ± 0.14	27	2.96 ± 2.24	*p* < 0.001	*p* = 0.006	*p* = 0.55
	no change	65	6.40 ± 0.35	163	3.44 ± 0.22	16	2.25 ± 2.79	η^2^ = 0.105	η^2^ = 0.009	η^2^ = 0.003
	increased	26	6.77 ± 0.56	64	4.34 ± 0.35	5	1.60 ± 1.52			
Communication with family	decreased	51	8.20 ± 0.39	90	5.57 ± 0.30	7	3.14 ± 1.95	*p* < 0.001	*p* = 0.021	*p* = 0.73
	no change	105	6.54 ± 0.27	274	4.09 ± 0.17	26	2.69 ± 2.57	η^2^ = 0.137	η^2^ = 0.007	η^2^ = 0.002
	increased	88	6.77 ± 0.30	242	4.57 ± 0.18	15	2.13 ± 2.29			
Leisure and Activity	decreased	182	7.19 ± 0.21	423	4.88 ± 0.14	32	2.69 ± 2.47	*p* < 0.001	*p* = 0.011	*p* = 0.160
	no change	54	6.59 ± 0.38	154	3.75 ± 0.23	13	2.69 ± 2.39	η^2^ = 0.038	η^2^ = 0.010	η^2^ = 0.003
	increased	8	4.50 ± 1.00	29	2.93 ± 0.52	3	1.00 ± 1.00			
Exercise	decreased	109	7.54 ± 0.27	278	4.91 ± 0.17	13	3.15 ± 2.23	*p* < 0.001	*p* = 0.011	*p* = 0.84
	no change	89	6.54 ± 0.30	229	4.10 ± 0.19	20	1.95 ± 2.28	η^2^ = 0.133	η^2^ = 0.007	η^2^ = 0.001
	increased	46	6.46 ± 0.42	99	4.28 ± 0.29	15	2.93 ± 2.60			
Food intake	decreased	25	7.88 ± 0.56	33	5.27 ± 0.49	2	2.00 ± 2.83	*p* < 0.001	*p* = 0.020	*p* = 0.86
	no change	116	6.45 ± 0.26	417	4.23 ± 0.14	38	2.34 ± 2.16	η^2^ = 0.075	η^2^ = 0.007	η^2^ = 0.001
	increased	103	7.34 ± 0.28	156	5.05 ± 0.23	8	3.88 ± 3.18			
Sleep duration	decreased	34	8.91 ± 0.47	49	6.14 ± 0.40	5	3.20 ± 2.17	*p* < 0.001	*p* = 0.002	*p* = 0.48
	no change	118	6.49 ± 0.25	385	4.06 ± 0.14	30	2.43 ± 2.47	η^2^ = 0.107	η^2^ = 0.012	η^2^ = 0.003
	increased	92	6.87 ± 0.29	172	5.01 ± 0.21	13	2.69 ± 2.39			
Alcohol consumption	no consumption	77	6.58 ± 0.33	194	4.80 ± 0.20	17	2.65 ± 2.85	*p* < 0.001	*p* = 0.33	*p* = 0.137
	decreased	37	6.97 ± 0.47	111	5.00 ± 0.27	11	2.45 ± 2.11	η^2^ = 0.019	η^2^ = 0.004	η^2^ = 0.002
	no change	72	7.14 ± 0.34	196	3.82 ± 0.20	11	1.91 ± 1.87			
	increased	58	7.28 ± 0.37	105	4.69 ± 0.28	9	3.44 ± 2.40			

GHQ: General Health Questionnaire, SOC: Sense of coherence, SD: Standard deviation; SOC levels: Weak SOC (SOC-13 ≤ 46); Moderate SOC (46 < SOC-13 < 72); Strong SOC (SOC-13 ≥ 72).

**Table 4:** Logistic regression analysis of the categories associated with poor mental health (GHQ-12), stratified by SOC level.

SOC Level	Weak SOC						Normal SOC						Strong SOC					
Variables	*n*	AOR	95% CI			*p* Value	*n*	AOR	95% CI			*p* Value	*n*	AOR	95% CI			*p* Value
Age	244	1.02	0.96–1.08			0.53	606	1.00	0.97–1.02			0.85	48	1.14	0.96–1.35			0.089
Gender																		
Female	156						339						18					
Male	88	0.58	0.24–1.36			0.62	267	0.63	0.43–0.91			0.101	30	32.51	0.91–1158.00			0.026
Living arrangements																		
With others	159						464						40					
Alone	85	1.29	0.47–3.53			0.21	142	1.48	0.92–2.36			0.015	8	0.03	0.00–7.94			0.180
Economic situation																		
Acceptable	135						415						33					
Not acceptable	109	1.06	0.46–2.42			0.89	191	1.42	0.95–2.12			0.080	15	4.88	0.33–72.27			0.22
Communication with friends																		
Decreased	153	0.9	0.35–2.35			0.83	379	2.41	1.59–3.65			<0.0001	27	1.21	0.12–11.96			0.87
No change	65						163						16					
Increased	26	1.07	0.26–4.45			0.93	64	1.20	0.63–2.27			0.58	5	0.30	0.00–30.20			0.59
Communication with family																		
Decreased	51	3.21	0.59–17.57			0.140	90	1.39	0.78–2.48			0.27	7	21.21	0.06–7039.53			0.29
No change	105						274						26					
Increased	88	0.79	0.33–1.89			0.59	242	1.24	0.82–1.87			0.31	15	0.58	0.04–8.57			0.70
Leisure and activity																		
Decreased	182	0.81	0.28–2.35			0.70	423	1.63	1.05–2.53			0.031	32	0.27	0.03–2.49			0.24
No change	54						154						13					
Increased	8	0.08	0.01–0.64			0.015	29	0.56	0.22–1.44			0.227	3	1.99 × 10^−8^^†^	n/a–n/a			0.39
Exercise																		
Decreased	109	2.05	0.74–5.66			0.16	278	1.05	0.69–1.61			0.81	13	9.68	0.54–172.21			0.100
No change	89						229						20					
Increased	46	1.11	0.35–3.49			0.86	99	1.12	0.63–2.00			0.69	15	1.40	0.04–46.64			0.85
Foods intake																		
Decreased	25	1.14	0.23–5.52			0.87	33	1.39	0.57–3.35			0.46	2	0.08	0.00–9.74			0.31
No change	116						417						38					
Increased	103	2.12	0.81–5.57			0.118	156	1.17	0.74–1.83			0.50	8	0.57	0.01–39.02			0.79
Sleep duration																		
Decreased	34	2.46 × 10^8^	n/a–n/a			0.004	49	3.39	1.45–7.93			0.002	5	3.24	0.08–137.91			0.54
No change	118						385						30					
Increased	92	1.2	0.48–3.04			0.69	172	1.39	0.91–2.13			0.131	13	404.15 *	2.67–61183			<0.001
Alcohol consumption																		
Non–drinker	77						194						17					
Decreased	72	1.59	0.43– 5.94			0.48	196	1.37	0.78–2.41			0.28	11	0.49	0.02–15.85			0.68
No change	37	1.6	0.57–4.46			0.37	111	0.61	0.40–0.96			0.030	11	0.67	0.03–16.16			0.80
Increased	58	1.79	0.56–5.73			0.32	105	0.93	0.54–1.61			0.80	9	5.08	0.30–87.01			0.24

SOC levels: Weak SOC (SOC-13 ≤ 46); Moderate SOC (46 < SOC-13 < 72); Strong SOC (SOC-13 ≥ 72). GHQ, General Health Questionnaire; SOC, Sense of coherence; OR, odds ratio; CI, confidence interval. ^†^ Low OR and lack of CI due to sample imbalance (i.e., all 3 people with increased leisure and activity defined healthy). * High OR and lack of CI due to sample imbalance (i.e., all 34 people with decreased sleep duration had poor mental health).
